# Subcortical structural connectivity of insular subregions

**DOI:** 10.1038/s41598-018-26995-0

**Published:** 2018-06-05

**Authors:** Jimmy Ghaziri, Alan Tucholka, Gabriel Girard, Olivier Boucher, Jean-Christophe Houde, Maxime Descoteaux, Sami Obaid, Guillaume Gilbert, Isabelle Rouleau, Dang Khoa Nguyen

**Affiliations:** 1 Département de psychologie, Université du Québec à Montréal, Montréal, Qc Canada; 2 Centre de Recherche du Centre Hospitalier de l’Université de Montréal, Montréal, Qc Canada; 3 BarcelonaBeta Brain Research Center, Pasqual Maragall Foundation, Barcelona, Spain; 4 Sherbrooke Connectivity Imaging Lab (SCIL), Computer Science department, Université de Sherbrooke, Sherbrooke, Qc Canada; 5 Département de psychologie, Université de Montréal, Montréal, Qc Canada; 6 Service de Neurochirurgie, Centre Hospitalier de l’Université de Montréal, Montréal, Qc Canada; 7 MR Clinical Science, Philips Healthcare, Cleveland, OH USA; 8 Service de Neurologie, Centre Hospitalier de l’Université de Montréal, Montréal, Qc Canada

## Abstract

Hidden beneath the Sylvian fissure and sometimes considered as the fifth lobe of the brain, the insula plays a multi-modal role from its strategic location. Previous structural studies have reported cortico-cortical connections with the frontal, temporal, parietal and occipital lobes, but only a few have looked at its connections with subcortical structures. The insular cortex plays a role in a wide range of functions including processing of visceral and somatosensory inputs, olfaction, audition, language, motivation, craving, addiction and emotions such as pain, empathy and disgust. These functions implicate numerous subcortical structures, as suggested by various functional studies. Based on these premises, we explored the structural connectivity of insular ROIs with the thalamus, amygdala, hippocampus, putamen, globus pallidus, caudate nucleus and nucleus accumbens. More precisely, we were interested in unraveling the specific areas of the insula connected to these subcortical structures. By using state-of-the-art HARDI tractography algorithm, we explored here the subcortical connectivity of the insula.

## Introduction

The insula is thought to play a role in various functions, including sensorimotor integration, olfaction, audition, language, processing of visceral sensations, motivation, craving, addiction, and emotions such as pain, disgust, empathy, happiness and anxiety^1,2^. Based on the results of a meta-analysis of functional neuroimaging studies, Kurth *et al*. classified these functions into four distinct groups: sensorimotor, olfacto-gustatory, socio-emotional, and cognitive functions^3^. This wide array of functions is subserved by its strategic location surrounded laterally by the frontal, temporal and parietal operculum, inside the Sylvian fissure, and medially by the extreme capsule and the claustrum^4^. The central sulcus divides the insula into an anterior and posterior sulco-gyral region, while its cytoarchitectonic composition divides it into an anterior agranular, intermediate dysgranular and posterior granular zone conditional to the organization, shape and type of neurons present^5^.

Tracing studies in nonhuman primates have described connections of the insula with the frontal, temporal, and parietal lobes as well as with the thalamus, hippocampus, amygdala, and putamen^6–8^. More recently, diffusion magnetic resonance imaging (MRI) using tractography reported similar cortical connections as those from nonhuman primates^9–11^. As for subcortical regions, only connections to the thalamus^11^, amygdala^9^ and putamen^10^ have been found in some participants or when using a low threshold of fibers. Latter studies have used a ball & stick or a constrained spherical deconvolution approaches. These techniques, alone, can recover local crossing fibers but are generally not designed to appropriately model partial volume caused by complex white matter crossing fiber pathways (such as the insula) and increase the risks of missing connections. In such regions, it is recommended to use: (1) anatomically-constrained tracking, which uses tissue information to end tracking in white-grey matter interface; (2) particle filter tractography based on prior anatomical tissue partial volume estimation (PVE) maps to decrease the number of broken fibers; (3) backtracking which incrementally truncates and re-tracks the streamline when it reaches a premature stop; (4) and tissue PVE maps to decrease regions of partial volume effects^12,13^. On the other hand, resting-state functional MRI connectivity studies have shown insular co-activation with the thalamus, amygdala, and hippocampus^14^.

By using state-of-the-art high angular resolution diffusion weighted imaging (HARDI) deterministic tractography based on constrained spherical deconvolution and particle filter tractography (PFT) with anatomical priors^15^, our team recently described in details the corticocortical connectivity profile of the insula, reporting more connections than previous studies, notably with the anterior and posterior cingulate gyri, the angular and lingual gyri as well as the precuneus and occipital lobe^16^. The methodology used led to a more precise estimation of fiber trajectories when facing crossing fibers in white matter bundles by analyzing the convergence of fiber bundles and thus, minimizing spurious streamlines and conveying better confidence regarding their density^12,15^. The fact that these structural connections had previously been inferred by functional studies in humans^14,17^ and identified in nonhuman primates using tracing techniques^8,18^ suggested that our tractography pipeline was viable. The absence of salient connections between the insula and subcortical regions in previous tractography studies in humans as well as the lack of noteworthy results in nonhuman primates led us to further examine this avenue. In this context, we used a streamline deterministic tractography algorithm combined with the PFT algorithm, to reduce premature stopping of streamlines^15^, on 46 healthy subjects to explore the structural connectivity of 19 distinct subinsular regions of interests (ROIs) with the following subcortical structures: the thalamus, putamen, hippocampus, globus pallidus, caudate nucleus, amygdala, and nucleus accumbens.

## Results

**Figure 1:**
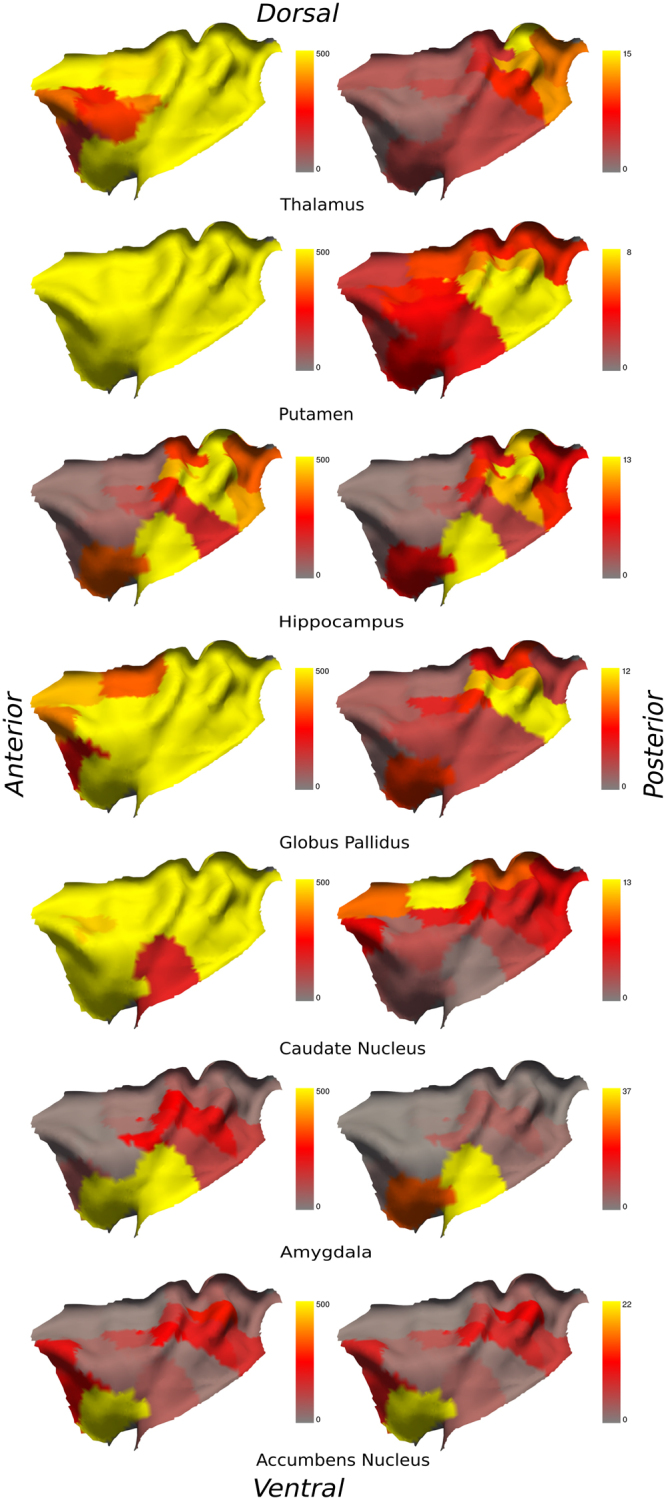
Left column: connectivity between the left insula and subcortical ROIs with a threshold ranging from 50 (red), 150 (orange) to 500 (yellow) tracts per voxel; Right column: percentage of the total fibers connecting every single ROIs of the left insula. From top to bottom: thalamus, putamen, hippocampus, globus pallidus, caudate nucleus, amygdala and nucleus accumbens.

**Figure 2:**
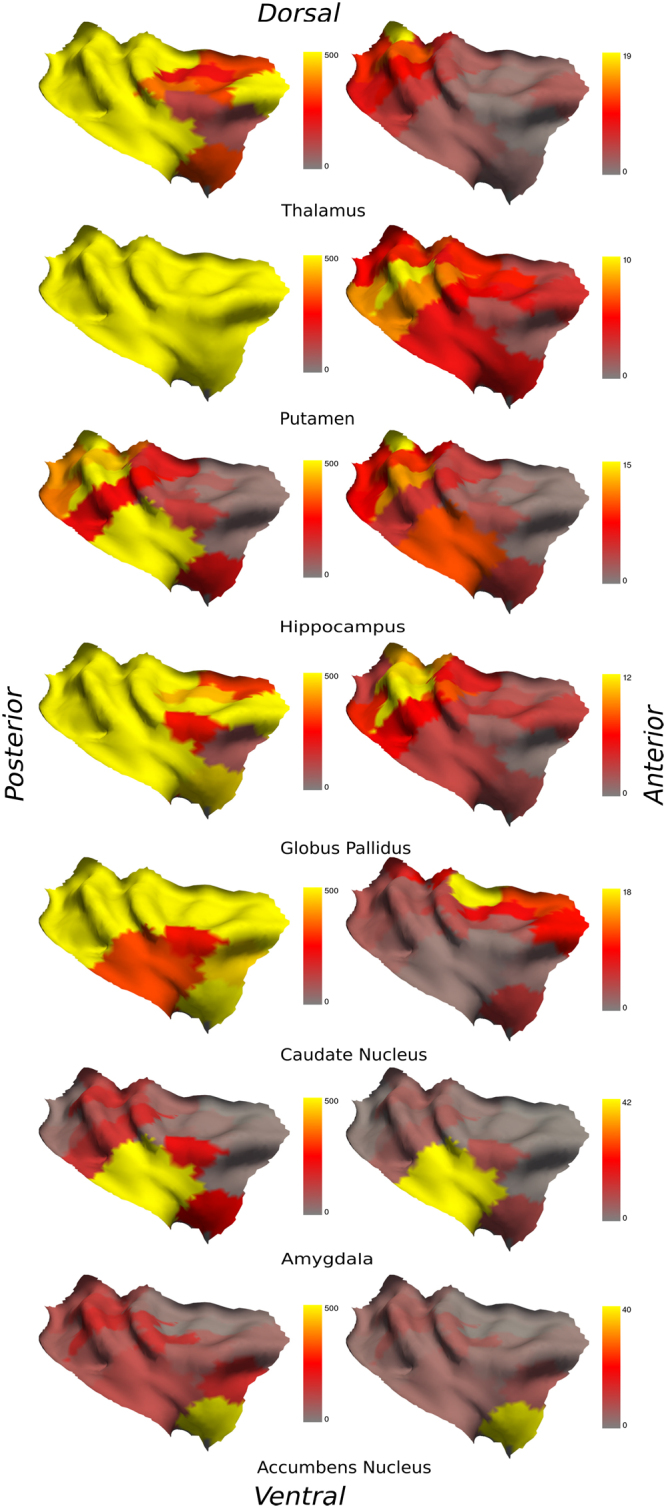
Left column: connectivity between the right insula and subcortical ROIs with a threshold ranging from 50 (red), 150 (orange) to 500 (yellow) tracts per voxel; Right column: percentage of the total fibers connecting every single ROIs of the right insula. From top to bottom: thalamus, putamen, hippocampus, globus pallidus, caudate nucleus, amygdala and nucleus accumbens.

The connectivity maps, which represents the bidirectional connectivity between insular and subcortical ROIs, ranging from 0 to 500 fibers and more streamlines per voxel, is illustrated in Fig. 1 (left hemisphere) and Fig. 2 (right hemisphere). These figures also depict the percentage of the total fibers connecting every single ROIs of the insula. The number of fibers and their corresponding percentages are represented in Table 1 (right hemisphere) and Table 2 (left hemisphere). Our results show that both insulae have connections with the seven subcortical regions examined.

**Table 1:** Connectivity between the ROIs of the left insula and the left subcortical ROIs with a threshold of 150 fibers per voxel.

ROIs	Thal	**%**	Put	**%**	Hipp	**%**	Glob	**%**	Caud	**%**	Amyg	**%**	Accu	**%**
1	3444	16.29%	11044	4.72%	677	11.99%	1844	7.02%	2689	8.89%	**43**	0.98%	**70**	2.63%
2	974	4.61%	10474	4.48%	309	5.47%	1359	5.17%	2718	8.98%	**71**	1.61%	**50**	1.88%
3	2382	11.27%	10899	4.66%	346	6.13%	826	3.14%	1784	5.90%	**29**	0.66%	**48**	1.81%
4	2724	12.89%	16695	7.14%	783	13.86%	2850	10.84%	1611	5.33%	**73**	1.66%	276	10.39%
5	1095	5.18%	14616	6.25%	436	7.72%	2825	10.75%	1583	5.23%	249	5.65%	**140**	5.26%
6	486	2.30%	11908	5.09%	**45**	0.80%	348	1.32%	4502	14.88%	**31**	0.70%	**14**	0.54%
7	547	2.59%	5127	2.19%	**30**	0.54%	442	1.68%	2842	9.39%	**16**	0.37%	**19**	0.73%
8	2568	12.15%	23201	9.92%	422	7.47%	3517	13.38%	1597	5.28%	**126**	2.85%	160	6.03%
9	1836	8.69%	22815	9.76%	647	11.46%	3334	12.68%	1430	4.73%	214	4.85%	195	7.31%
10	628	2.97%	17688	7.57%	282	4.99%	1821	6.93%	2014	6.66%	221	5.02%	239	8.98%
11	667	3.15%	10048	4.30%	**81**	1.43%	1129	4.29%	1529	5.05%	**45**	1.03%	**106**	4.00%
12	287	1.36%	10841	4.64%	**38**	0.67%	503	1.91%	462	1.53%	**11**	0.25%	**54**	2.04%
13	364	1.72%	6438	2.75%	**29**	0.52%	386	1.47%	1887	6.24%	**18**	0.41%	212	7.98%
14	759	3.59%	22566	9.65%	172	3.05%	700	2.66%	762	2.52%	**118**	2.68%	**33**	1.23%
15	381	1.80%	8861	3.79%	**110**	1.95%	806	3.07%	725	2.40%	238	5.41%	**57**	2.15%
16	309	1.46%	8807	3.77%	**54**	0.95%	734	2.79%	810	2.68%	**18**	0.40%	**50**	1.87%
17	163	0.77%	4823	2.06%	**48**	0.85%	229	0.87%	615	2.03%	**80**	1.82%	236	8.86%
18	747	3.53%	7683	3.29%	794	14.05%	737	2.80%	183	0.61%	1699	38.52%	**77**	2.89%
19	778	3.68%	9274	3.97%	345	6.10%	1899	7.22%	509	1.68%	1109	25.13%	623	23.40%
Total	21138	100.00%	233807	100.00%	5649	100.00%	26288	100.00%	30253	100.00%	4412	100.00%	2661	100.00%

Regions with less than 150 fibers per voxel are in bold.

**Table 2:** Connectivity between the ROIs of the right insula and the right subcortical ROIs with a threshold of 150 fibers per voxel.

ROIs	Thal	**%**	Put	**%**	Hipp	**%**	Glob	**%**	Caud	**%**	Amyg	**%**	Accu	**%**
1	4424	20.99%	10477	5.24%	826	16.39%	2034	10.33%	2314	6.51%	**109**	3.78%	**109**	4.07%
2	1498	7.10%	13038	6.52%	364	7.23%	1903	9.67%	3347	9.42%	**126**	4.37%	**88**	3.27%
3	2312	10.97%	10238	5.12%	403	8.00%	632	3.21%	1177	3.32%	**25**	0.88%	**94**	3.52%
4	2726	12.93%	11417	5.71%	454	9.01%	2258	11.47%	908	2.56%	156	5.44%	**134**	4.99%
5	879	4.17%	8707	4.36%	166	3.29%	1076	5.47%	1354	3.81%	**113**	3.92%	**40**	1.48%
6	584	2.77%	11413	5.71%	194	3.84%	897	4.56%	6788	19.11%	**55**	1.91%	**24**	0.88%
7	321	1.52%	4467	2.24%	**29**	0.58%	308	1.57%	4069	11.46%	**6**	0.21%	**21**	0.80%
8	1722	8.17%	16215	8.11%	377	7.47%	1354	6.88%	950	2.67%	**40**	1.39%	**71**	2.66%
9	2098	9.95%	23727	11.87%	619	12.28%	2716	13.80%	1030	2.90%	**94**	3.27%	162	6.05%
10	821	3.89%	15047	7.53%	264	5.24%	1588	8.07%	1603	4.51%	167	5.80%	**140**	5.20%
11	218	1.03%	11193	5.60%	**69**	1.37%	439	2.23%	2914	8.21%	**27**	0.93%	**12**	0.45%
12	323	1.53%	8277	4.14%	**34**	0.67%	648	3.29%	945	2.66%	**26**	0.91%	**61**	2.28%
13	603	2.86%	6455	3.23%	25	0.50%	590	3.00%	3374	9.50%	**15**	0.54%	**47**	1.75%
14	1129	5.35%	15790	7.90%	229	4.54%	1152	5.86%	681	1.92%	151	5.24%	**85**	3.15%
15	367	1.74%	9729	4.87%	**103**	2.04%	685	3.48%	1175	3.31%	**39**	1.36%	**145**	5.41%
16	**122**	0.58%	3907	1.96%	140	2.77%	270	1.37%	270	0.76%	218	7.59%	**71**	2.63%
17	**91**	0.43%	2451	1.23%	**29**	0.57%	**81**	0.41%	465	1.31%	**17**	0.61%	166	6.17%
18	524	2.48%	8488	4.25%	498	9.88%	572	2.91%	323	0.91%	1236	43.03%	**106**	3.95%
19	319	1.51%	8796	4.40%	217	4.31%	476	2.42%	1827	5.15%	253	8.82%	1108	41.30%
Total	21080	100.00%	199831	100.00%	5040	100.00%	19681	100.00%	35515	100.00%	2872	100.00%	2683	100.00%

Regions with less than 150 fibers per voxel are in Bold.

### Thalamus

The left thalamus is connected with every single ROIs of the left insula, as for the right insula. We did not find connections with rostral mid-anterior ROIs when considering a threshold of 150 fibers per voxel. These ROIs appear connected with less than 100 fibers per voxel. The ROI most connected to the left thalamus (with 16% of total fibers) was the ipsilateral ROI 1 located in the dorsal posterior insula. The ROI most connected to the right thalamus (with 21% of total fibers) was the ipsilateral ROI 1 as well, also located in the dorsal posterior insula.

### Putamen

The left and the right putamen are fully connected to the ipsilateral insula ROIs. These connections are still observed with a threshold of 500 fibers per voxel. The insular ROI showing the most connections to the left and right putamen was the ipsilateral ROI 8 located in the ventral posterior insula (10% and 8% of total fibers, respectively).

### Hippocampus

The left and right hippocampi have somewhat symmetrical connections with the left and right ventral and dorsal posterior insular ROIs. A similar symmetry is observed with more than 150 fibers per voxel for mid-ventral and mid-dorsal insular ROIs in both hemispheres. The most connected insular ROI to the left hippocampus was the ipsilateral ROI 18 in the ventral intermediate part of the insula (14% of total fibers); for the right hippocampus, it was the ipsilateral ROI 1 in the dorsal posterior insula (16% of total fibers).

### Globus pallidus

The left globus pallidus is fully connected to the left insula; as for the right insula, we did not find connections with mid-anterior ROIs. On the other hand, this part seems connected with less than 100 fibers per voxel. The most connected ROI to the left globus pallidus was the ipsilateral ROI 8 in the ventral posterior insula (13% of total fibers). The most connected ROI to the right globus pallidus was the ipsilateral ROI 9 in the ventral posterior insula (14% of the total fibers).

### Caudate nucleus

The left and the right caudate nuclei are fully connected to the left and right insula’s ROIs respectively. These connections are still observed with 500 fibers per voxel. The most connected ROI to the left caudate nucleus was the ipsilateral ROI 6 in the dorsal anterior insula (15% of total fibers); the most connected ROI to the right caudate nucleus was the ipsilateral ROI 6 as well, also in the dorsal anterior insula (20% of total fibers).

### Amygdala

The left amygdala is mostly connected to the ventral and intermediate anterior, and mid-posterior ROIs of the left insula, while the right amygdala has connections with ventral anterior and posterior ROIs of the right insula. The left and right amygdala have connections with less than 100 fibers per voxel with dorsal posterior ROIs of the left and right insula. The insular ROI showing the higher proportion of fibers connected to the left amygdala was the ipsilateral ROI 18 in the ventral intermediate part of the insula (39% of total fibers); the most connected ROI to the right amygdala was the ipsilateral ROI 8 in the ventral posterior insula (43% of total fibers).

### Nucleus accumbens

The left nucleus accumbens is connected with ventral to slightly dorsal anterior, as well as some dorsal posterior left insula ROIs; the right nucleus accumbens is connected with ventral anterior ROIs and some mid-dorsal posterior ROIs of the right insula. With a threshold of less than 100 fibers per voxel however, both left and right nucleus accumbens have connections with every ROIs of the posterior regions of the left and right insulae. The most connected ROI to the left nucleus accumbens was with the ipsilateral ROI 6 in the dorsal anterior insula (9% of total fibers); the most connected ROI to the right nucleus accumbens was also the ipsilateral ROI 6 in the dorsal anterior insula (19% of total fibers).

## Discussion

Our work reveals a rich insular connectivity pattern with subcortical structures. The majority of connections have more than 150 fibers per voxels and remain stable even at a threshold of 500 fibers or more per voxel. This threshold was used to ensure reliable, dense and non-spurious fiber bundles connecting ROIs. Moreover, we observe a relatively symmetrical connectivity profile between the two hemispheres. Our state-of-the-art PFT tractography algorithm on HARDI diffusion data upsampled to 1 mm, with probabilistic maps acting as anatomical priors, may be responsible for observing these findings because it allows a better propagation in narrow and tight white-matter bundles present around the insula and when entering subcortical regions^13,15^. Indeed, a proportion of connections reconstructed from tractography is biased by the position, the shape, the size and the length of white matter fascicles^15,19–22^. Therefore, measures of connectivity based on streamlines distribution in the brain such as streamline count or density are biased by erroneous streamlines produced by tractography algorithms. PFT uses anatomical information derived from a high resolution T1-weighted image to enforce the connection of streamlines to gray matter regions and to reduce biases in the distribution of streamlines^15^. Consequently, PFT algorithm allowed us to obtain more robust results than previous studies regarding partial volume effects and broken fibers (e.g. streamlines that stop prematurely in the white matter), which is particularly crucial for tracking a deep structure such as the insula.

The literature mainly reports connections with subcortical regions in relation to specific pathologies, while few studies have looked into the healthy subcortical connectivity of the insula. We report connections with the thalamus, hippocampus, amygdala and putamen in accordance with prior nonhuman primate tracing studies^6–8,18^ and with the thalamus, amygdala, hippocampus, and putamen in accordance with prior human tractography studies^9–11,23,24^. Indeed, Nomi *et al*.^24^ recently reported connectivity with a threshold of 1 tract per voxel in at least 75% of the participants in one insular ROI and in one hemisphere for the thalamus (left dorsal anterior insula) and the hippocampus (right ventral anterior insula). Wiech *et al*.^23^ also reported connectivity with the thalamus and amygdala based on 100 seeds per voxel with both anterior and posterior insular regions. Aside from these regions, we reveal additional structural connections with the nucleus accumbens, caudate nucleus and globus pallidus. In the following paragraphs, we consider insular connections of each subcortical structure mentioned above separately and discuss their possible contribution to the various roles of the insula.

### Thalamus

The thalamus is a strategic and major structure of the brain. It works as a relay station for every sensory input – with the exception of olfactory inputs – to the cerebral cortex, and is also involved in several functions including arousal and alertness, memory, autonomic functions, and gaze control^25–27^. It has widespread functional connections across cortical and other subcortical areas^28–30^. Congruent with previous studies in animals and humans, we found connections between the thalamus and the insular lobe^7,14,31^. All ROIs in the right insula, and most in the left insula, showed connections with the thalamus. Connections between the thalamus and the anterior insula may underlie processing of information related to gustatory, visceral, and autonomic functions as well as of salient information and emotional processes, whereas connections with the posterior insula may be related to auditory and somatosensory processing^7,32^.

### Putamen

The putamen, along with the caudate nucleus, forms the dorsal striatum. The role of the putamen in motor processes and, consequently, in the motor manifestations of Parkinson’s disease, is well established^33,34^. The putamen is also thought to be involved in instrumental learning and in somatosensory processing, especially pain^33,35^. Resting-state fMRI has previously revealed functional connections between the caudal putamen and primary and supplementary cortical motor areas, congruent with its role in motor function, and connections between the rostral putamen and the dorsolateral prefrontal cortex and anterior cingulate cortex, associated with executive control^36^. The ventral rostral putamen was also shown to be connected with the insular cortex^36,37^. In the present study, we reported putaminal connections with every ROIs of the left and right insula. Functional studies reported the dorsal anterior insula to be involved in several functions^3^ including speech production^38^ and pain processing^39^, and may also play a role in drug addiction^40,41^ and in non-motor manifestations of Parkinson’s disease such as somatosensory and autonomic disturbances, cognitive impairments and behavioral changes^42^. Additionally, the rich connectivity between the insula and the putamen may be explained by its anatomical proximity, as these regions are only separated by the extreme and external capsules^4^.

### Hippocampus

The hippocampus is crucial for episodic and spatial memory^43,44^. Resting-state fMRI in normal adults has revealed extensive functional connectivity with cortical and limbic regions^45^. We observed connections between the hippocampus and the anterior and posterior insula. Interestingly, a study using electrical cortical stimulation in epileptic patients recorded reproducible evoked potentials in the inferior portion of the insula, more consistently in the posterior insula, 23 to 138 ms after stimulation of the hippocampus, while evoked potentials in the superior part of the insula occurred later and were less consistent^46^. The posterior insula has been associated with sensori-motor processing and vestibular function^47^, and connections between this region and the hippocampus may facilitate navigation and spatial learning^48^. Functional studies have shown an implication of both dorsal anterior insula in working memory tasks such as n-back and Sternberg paradigms, as well as episodic and short-term memory retrieval^3^. Connections with the ventral anterior insula, which has been associated with socio-emotional processing^3^, may participate to the mediation of memory encoding by emotionally arousing information^49^. Insular-hippocampal connections may also account for certain symptoms associated with epileptic seizures originating from the hippocampus, such as viscerosensory and olfactory-gustatory auras^50^.

### Globus pallidus

The globus pallidus has been involved in a variety of speech functions, some of which may be intimately related to the insula^51,52^. The left anterior insula has been linked to speech production and articulatory processing^3,53,54^, and left insular damage following ischemic lesions may result in apraxia of speech and dysarthria^55^. The globus pallidus seems to be involved in temporal synchronization of linguistic modules^51^. We observed multiple connections between the whole left insula and the globus pallidus. Functional connectivity between the internal globus pallidus and the left ventral anterior to middle insula has been observed within the speech network^52^. Since the left anterior and middle insula have been associated with emotional processing and sensorimotor function respectively^52^, it is conceivable that such integration may be in part permitted by pallido-insular connections. Interestingly, insular hypoperfusion has been described in patients with speech disturbances from Parkinson’s disease^56^. Indeed, patients with Parkinson’s disease may develop abnormal speech fluency, dysarthria or hypophonia, all of which are thought to be related to dysfunctional basal ganglia^52^. Whether pallido-insular connectivity plays a role in the development of speech disturbances in Parkinson’s disease remains uncertain, but the limited improvement of speech functions following dopamine supplementation suggests a pathological process beyond the basal ganglia which may involve structurally connected cortical regions such as the insula.

### Caudate nucleus

The caudate nucleus plays a key role in many associative, executive, motivational, and affective processes^36,57^. Accordingly, functional and structural abnormalities within the caudate nucleus have been observed in dyscognitive pathologies such as psychosis, schizophrenia, obsessive-compulsive disorder and attention-deficit/hyperactivity disorder^36,57,58^. Functional imaging studies have revealed a prominent role of the dorsal anterior insula in cognition, attention, and decision-making while the ventral anterior insula was involved in emotional processes^3,59^. Interestingly, we observed extensive bilateral structural connections between two functionally related areas, namely the caudate nucleus and the anterior insula. Moreover, a meta-analytic functional connectivity study revealed bilateral connection of the caudate nucleus with the insula^60^, further supporting that the shared functions of the two regions likely result from underlying structural connections. The insula is an important region involved in pain perception, showing consistent activation in response to noxious stimuli in neuroimaging studies^61,62^. In addition, bilateral direct cortical stimulation of the posterior insula in patients undergoing invasive monitoring has been shown to elicit painful sensations^47,63^. Functional imaging studies have linked the caudate nucleus to affective processing and suppression of pain^64,65^. We found bilateral structural connections between the caudate nucleus and the whole surface of the insula. Accordingly, extensive functional connections between the caudate nucleus and the anterior insula have been observed during painful tasks^65^. The central role of the insula and the caudate nucleus in pain processing may therefore underlie the strong connectivity between these two functionally complementary areas.

### Amygdala

The amygdala is part of the limbic system and has been largely studied for its role in fear processing, including fear experience, fear conditioning, and recognition of fearful expressions^66–68^. Besides fear, it is also involved in other emotional functions, such as reward processing and motivation, and modulates attention, perception, and memory according to the emotional significance of external stimuli^69^. Functional connectivity of the amygdala, studied with resting-state fMRI, has been shown with the medial prefrontal cortex, insula, thalamus, and striatum^28^. Connectivity between the anterior insula and basolateral amygdala has been found to be strongly correlated with state anxiety^70^. The amygdala and anterior insula, especially the ventral part, share many functional characteristics, are both commonly activated by emotional stimuli^71^ and during risky decisions^72^ in neuroimaging studies, and have both been proposed to be part of a brain system integrating interoception, emotion, and social cognition^73^. Interoception, imagination and recall of one own emotions have been reported to activate bilateral ventral anterior insula in functional studies^3^. Based on this evidence, it is thus not surprising that connections were observed between these two regions. The lack of connectivity between the amygdala and the anterior regions of the insula may be due to the posterior location of the amygdala, thus making it harder for fibers to reach it through deep and crossing white matter fasciculus.

### Nucleus accumbens

The nucleus accumbens is part of the ventral striatum and plays a crucial role in motivational and emotional processes. It is considered as a limbic-motor interface, evaluating rewarding contexts directing attention and behavior towards positive stimuli such as food, sex and drugs, while avoiding aversive consequences. Aside from its role in novel stimuli processing and novel experiences, it has been reported as being implicated in multiple neurological and psychiatric disorders, such as depression, anxiety disorder, obsessive-compulsive disorder, bipolar disorder, Parkinson’s disease, Alzheimer’s disease, Huntington’s disease, obesity and addiction to drugs^74,75^. Functional connectivity of the nucleus accumbens have shown a role in locomotion learning, avoidance, impulsivity, risk-taking behaviors, feeding behavior, sexual motivation, incentive and reward (for a review see Salgado *et al*.^74^). We observed connections with the nucleus accumbens and the ventral anterior and dorsal posterior bilateral insula. Since no connections between the insula and the nucleus accumbens had previously been reported in the literature, we can only hypothesize a linked role of these regions in impulsivity^76,77^, emotion processing^3^, addiction^41,78–80^ risky decisions^81,82^ and reward circuitry^83^, Tourette syndrome, depression, bipolar disorder, anxiety disorder, Huntington’s and Alzheimer’s diseases^1,74,84^.

### Limitations

The main limitation of the current study, as mentioned in our previous study^16^, is of technical nature, related to the tractography approach. While it is the most appropriate noninvasive *in-vivo* investigative method in humans^85^, its precision is limited by the resolution of the images making it difficult to correctly estimate the trajectory of crossing-fibers, especially in subcortical regions where fiber bundles are denser. Thus, it remains indispensable to investigate brain pathology and explore the relationship between healthy and pathological connectivity and measure changes in aging in white matter architecture^86^. Moreover, tractography remains in essence an indirect measure of connectivity. Maier-Hein *et al*.^19^ mentioned that although most proposed algorithms are able to produce tractograms containing 90% of ground truth bundles simulations, reproducibility or prediction errors evaluations cannot validate the accuracy of reconstruction due to the lack of ground truth information in humans^19^. Henceforth, the main challenge is our limited knowledge of the anatomy to reconstruct. False-positives are still present, and the rate does not seem to change when using more robust parameters such as the maximal angular precision of the signal. The insula is surrounded by many close structures such as the claustrum and putamen, which may take up most of the connections making it harder to estimate properly connections in deeper regions such as the globus pallidus, hippocampus, amygdala, nucleus accumbens and thalamus. Hence, we cannot rule out that some of the connections to the insula might be spurious and have only been reported because of the proximity of subcortical regions compared to widespread cortical structures. This may possibly explain the lack of reported connections with some parts of these regions. Therefore, to reduce the risks of false positives, we used a deterministic PFT algorithm with anatomical priors instead of a probabilistic one^87–89^. Furthermore, the absence of standard criteria in diffusion algorithms and preprocessing methods may affect the outcome between studies^90^. The anatomical accuracy of tractography is highly dependent on the parameters used, such as the type of diffusion model, the angular threshold and the composition of the seed ROI. The use of inappropriate or ill-adapted parameters may lead to contaminated results leading to the omission or the overestimation of connections between structures (false-positives/false-negatives). Moreover, the choice of parameters that produces the best combination of sensitivity and specificity varies for different pathways^86^. Hence, one should select the parameters best suited for the objective of the study, as differences may still occur, even though current diffusion modeling techniques successfully recover up to 77% of valid bundles^19^. Additionally, we cannot distinguish between afferent and efferent projections with diffusion images, unlike tract-tracing injection techniques^91^. Finally, it is possible that a few voxels of ventro-posterior ROIs of the insula include parts of the claustrum and that a few voxels of ventro-rostral anterior ROIs of the insula include parts of the orbitofrontal cortex because of the limited resolution of MRI data.

In this study, we report a comprehensive connectivity profile of 19 insular ROIs with subcortical structures. In accordance with the limited literature in nonhuman primates and humans, we report connections with the putamen, the thalamus and the amygdala. We further reveal clear connections with the caudate nucleus, nucleus accumbens, globus pallidus, and hippocampus. Our results provide a structural basis to fundamental functions such as viscerosensory and sensorimotor processing, olfaction, audition, language, motivation, craving, addiction, memory and emotions. The fast improvement of tractography algorithms and novel segmentation techniques will further aid the exploration of insular connections to specific nuclei of these subcortical regions.

## Materials and Methods

### Participants

Forty-six healthy right-handed subjects between the age of 19 and 39 years old (mean age 24 years, SD 4.8; 28 women), with no history of neurological or psychiatric disorders, were recruited. Informed written consent was obtained from all participants for procedures approved by the Centre Hospitalier de l′Université de Montréal (CHUM) ethics board, in accordance with the latest revision of the declaration of Helsinki.

### Data Acquisition

MRI data were acquired on a 3 T Achieva X scanner (Philips, the Netherlands). The diffusion-weighted images were acquired with a single-shot spin-echo echo-planar pulse sequence (TR = 7.96 ms; TE = 77 ms; flip angle = 90°; slices = 68; field of view = 230 mm; matrix = 128 × 128; voxel resolution = 1.8 × 1.8 × 1.8 mm; readout bandwidth = 19.6 Hz/pix*els; echo-planar imaging direc*tion bandwidth = 1572.5 Hz; 8-channel head coil; SENSE acceleration factor = 2). One pure T2-weighted image (b = 0 s/mm^2^) and 60 images with noncollinear diffusion gradients (b = 1500 s/mm^2^) were obtained. In addition, T1-weighted images were acquired using 3D T1 gradient echo (scan time = 8.11 min; TR = 8.1 ms; TE = 3.8 ms; flip angle = 8°; slices = 176; voxel size = 1 × 1 × 1 mm, FOV 230 × 230 mm).

### Anatomical Images Preprocessing

Anatomical T1-weighted images were processed with the FMRIB’s software library (FSL; http://fsl.fmrib.ox.ac.uk/fsl/fslwiki/FSL)^92–94^. Non-brain tissues were removed with the brain extraction tool (BET; Smith^95^). Resulting brain images were then segmented into probabilistic maps of white and gray matter, and cerebrospinal fluid for each subject, using FMRIB’s automated segmentation tool^96^.

### Creation of the insula and the subcortical Regions of Interests

**Figure 3:**
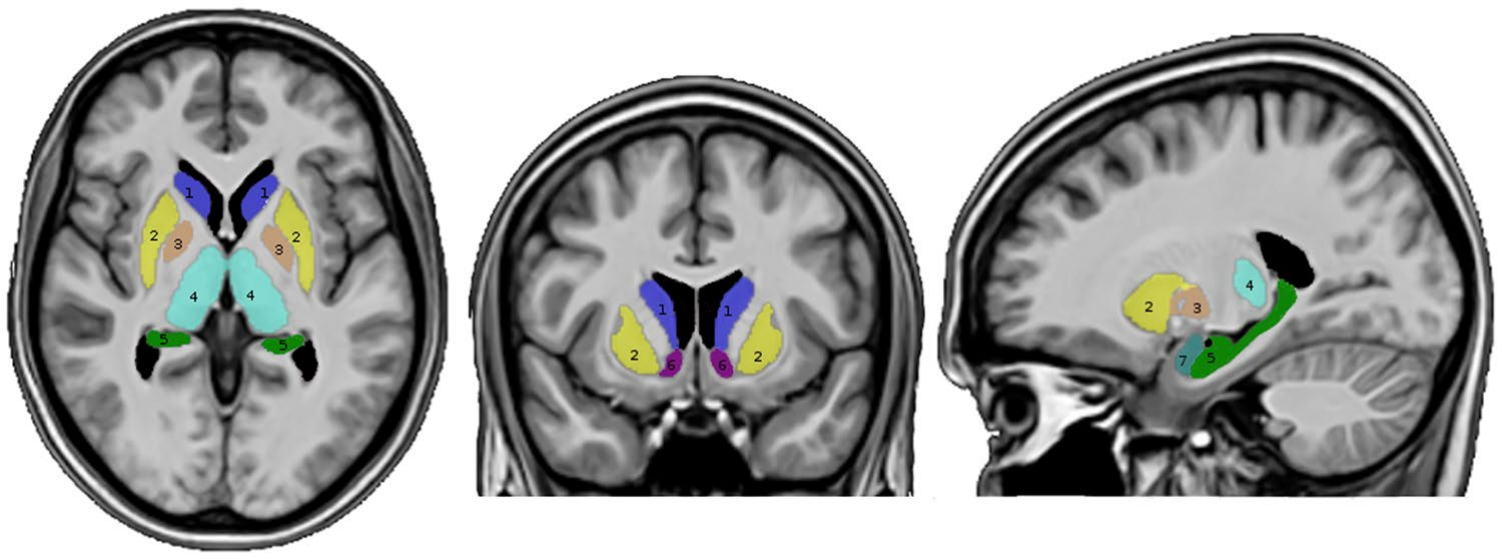
Segmented parcellation of the seven (7) subcortical ROIs: 1 (blue) = caudate nucleus, 2 (yellow) = putamen, 3 (tawny) = globus pallidus, 4 (turquoise) = thalamus, 5 (green) = hippocampus, 6 (purple) = nucleus accumbens, 7 (light blue) = amygdala.

**Figure 4:**
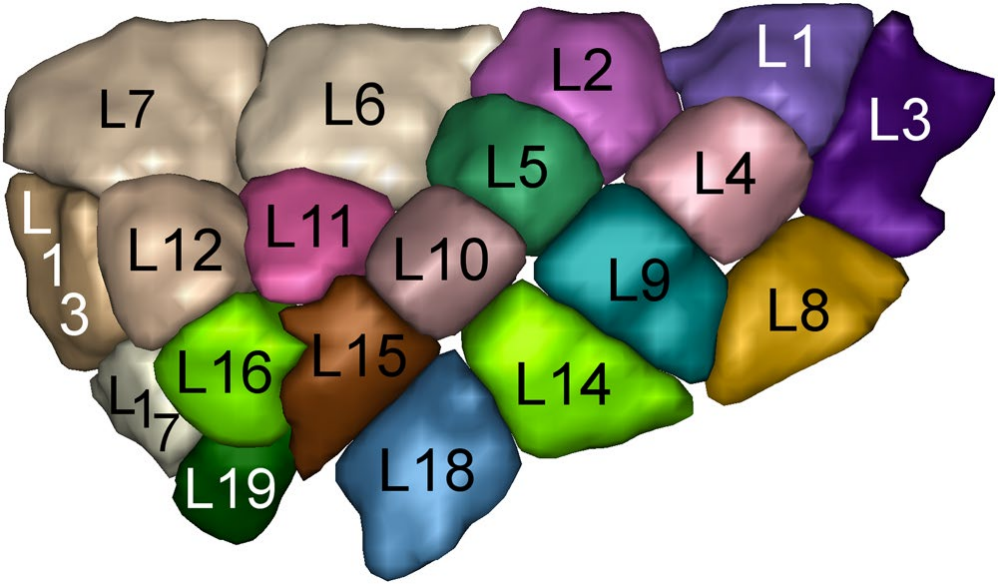
Sulco-gyral data-driven parcellation of the left insular cortex into 19 regions.

**Figure 5:**
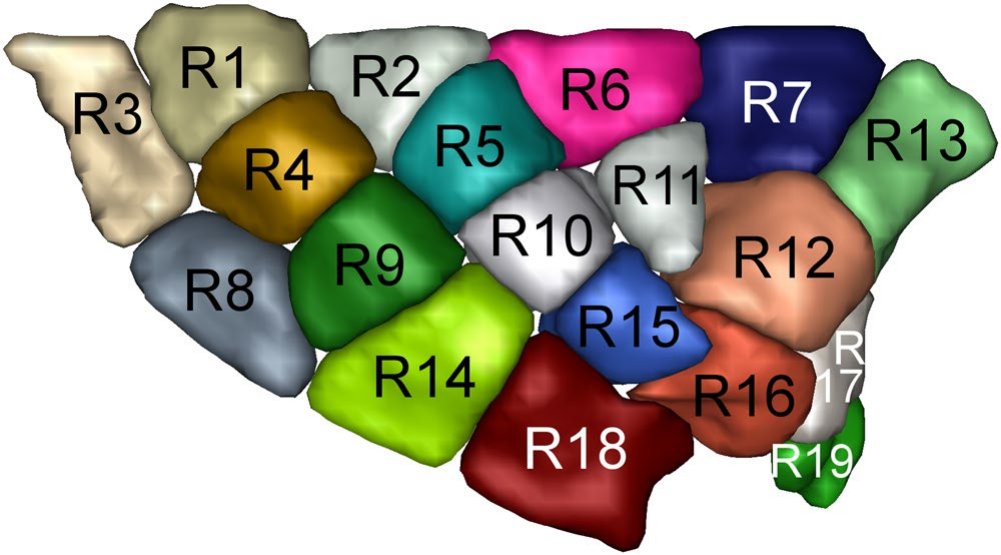
Sulco-gyral data-driven parcellation of the right insular cortex into 19 regions.

We used the volBrain online automated MRI brain volumetry system (http://volbrain.upv.es/)^97^ to obtain the segmentation of the thalamus, putamen, hippocampus, globus pallidus, caudate nucleus, amygdala and nucleus accumbens for every subject, individually (Fig. 3). The outputs were checked by two investigators to ensure the quality of the ROIs. Steps describing the segmentation of the insula are described in our previous work (Figs 4 and 5)^16^.

### Particle Filter Tractography with anatomical priors

HARDI data were first corrected for eddy current and head movement using FSL’s diffusion toolbox. Image quality was then increased using non-local means Rician de-noising method^98^. Resulting diffusion images were then up-sampled to a voxel size of 1 × 1 × 1 mm giving finer details on the tissue partial volume estimation maps to guide the white matter reconstruction using the PFT tractography algorithm^15,99^. This step allows to use the partial volume estimation maps derived from the T1-weighted image without down-sampling them to the diffusion images resolution (1.8 × 1.8 × 1.8 mm^3^). We used a white-matter probabilistic map, obtained from anatomical T1-weighted image, in the tracking algorithm as it has been shown to produce richer and more accurate streamlines than a thresholded FA map^15^. The co-registered probabilistic white matter map of the anatomical T1-weighted image was done with ANTs affine registration^100^. Similarly, the ROIs of the insula were resampled to every single-subject diffusion space. A detailed description, such as the steps to verify the validity of the registration, is available in our previous work^16^.

Constrained spherical deconvolution (CSD)^101,102^ computation was performed using MRtrix (v.0.2.12)^103^ prior to the streamline tracking algorithm on fiber orientation distribution functions (fODF). We then used the deterministic PFT parameters proposed in Girard *et al*.^15^ to reduce risks of reconstructing false positives pathways. We used a threshold of 150 seeds per voxel from all 19 ROIs of the insula and 7 subcortical regions in both hemispheres to obtain the maximum spatial extent of the bundles. PFT weighs the propagation pathways based on the partial volume estimation maps estimated from the T1-weighted image to enforce the tracking in the white matter. Propagation pathways are chosen to ensure that the streamlines do not stop in the CSF and reach the gray matter^15,22^. The PFT algorithm backtracks a short distance from an incorrect stopping event, then generates multiples probabilistic streamlines penalizing those that propagates in voxels containing partial volume of CSF. It simultaneously estimates many propagation pathways at a short distance of the premature stopping event to estimate a likely streamline. Finally, a streamline is drawn from the final estimated distribution of streamlines and the deterministic tractography algorithm restarts normally. Since diffusion MRI cannot differentiate between the afferent and efferent orientation of a fiber, the seeds were launched from the insula and the subcortical structures. The probabilistic subject’s grey matter subcortical map was used as an inclusion parameter, and the CSF and non-brain voxels as an exclusion parameter; the step size was 0.5 mm, as described in Girard *et al*.^15^. A more detailed description of the rationale behind the seeds and fibers threshold is available in our previous work^16^.

### Normalization

To account for differences in size between subcortical regions as well as insular ROIs, we normalized the number of fibers connecting insular and subcortical ROIs to the number of voxels of insular and subcortical ROIs. Because tractography was launched from al 19 ROIs of the insula to subcortical regions and vice versa, we then summed each connection with its inverse for a better estimation of the real connectivity between each pair of ROIs.
